# Spatially dependent atom-photon entanglement

**DOI:** 10.1038/s41598-018-32051-8

**Published:** 2018-09-14

**Authors:** Zahra Amini Sabegh, Rahim Amiri, Mohammad Mahmoudi

**Affiliations:** 1 Department of Physics, University of Zanjan, University Blvd., 45371-38791 Zanjan, Iran; 2 Young Researchers and Elite Club, Zanjan Branch, Islamic Azad University, Zanjan, Iran

## Abstract

The atom-photon entanglement using the Laguerre-Gaussian (LG) beams is studied in the closed-loop three-level *V*-type quantum systems. We consider two schemes with near-degenerate and non-degenerate upper levels: in the first, the effect of the quantum interference due to the spontaneous emission is taken into account and in the second, a microwave plane wave is applied to the upper levels transition. It is shown that the atom-photon entanglement in both schemes depends on the intensity profile as well as the orbital angular momentum (OAM) of the applied fields so that the various spatially dependent entanglement patterns can be generated by Laguerre-Gaussian beams with different OAMs. However, due to the zero intensity,no entanglement appears in the center of the optical vortex beams. As a result, the entanglement between dressed atom and its spontaneous emissions in different points of the atomic vapor cell can be controlled by the OAM of the applied fields. Moreover, our numerical results show that the number of the local maximum degree of entanglement (DEM) peaks depends on the OAM of the applied fields. The degrees of freedom for OAM play a crucial role in spatially dependent atom-photon entanglement in such a way that it may possess broad applications in high-dimensional quantum information processing and data storage.

## Introduction

Entanglement is generally a quantum mechanical phenomenon without any classical analogous which makes a correlation between the parts of a multi-partite system^1,2^. A quantum state of a bipartite entangled system cannot be described by a simple tensor product of quantum states of two subsystems^3^. Not only does atom-photon entanglement play an outstanding role in quantum mechanics such as early measurement of Bell inequality violations^4^, but it has also high potential applications in teleportation^5^, cryptography^6^, error correction^7^ and other quantum computation processes^8^.

Generally speaking, the angular momentum carried by light can be distinguished by the spin angular momentum associated with circular polarization^9^ and the orbital angular momentum (OAM) associated with the spatial distribution of the wavefront. In 1992, Allen *et al*. showed that the Laguerre-Gaussian (LG) laser mode has a well-defined OAM, as $l\hslash$ per photon, and proposed an experiment to observe the torque on cylindrical lenses arising from the reversal of the helicity of a LG mode^10^. An additional motivation for recent studies of LG beams is as a route to obtain narrower electromagnetically induced transparency (EIT) spectrum than the conventional Gaussian one^11–13^. The transmission of structured light has been measured using the phase profile in cold rubidium atoms and been shown that the EIT is spatially dependent for vortex light beams^14^. Recently, Mahmoudi *et al*. has investigated the effect of LG intensity profile on the optical spectrum of multi-photon resonance phenomena. It has been found that the linewidth of the optical spectrum due to the multi-photon transition becomes narrower in the presence of a LG beam^15^. They have also studied trap split in an atomic system interacting with femtosecond LG laser pulses^16^. More recently, we have demonstrated that the LG beam can decrease the full width at the half maximum of the output probe intensity in the electromagnetically induced focusing^17^.

It is well known that a LG light beam with an azimuthal phase dependence of ${e}^{-il\varphi }$ carries quantized OAM^18^. Due to the fact that the optical properties of the closed-loop quantum systems, in multi-photon resonance condition, depend on the relative phase of applied fields^19^ the effect of the OAM of the LG beams can be seen in this system due to the azimuthal phase factor of LG beam via the multi-photon transitions.

Increasing the complexity of entangled systems is important in the various applications of the entanglement in quantum information tasks which can be essentially done by increasing the number of particles involved in the entanglement^20^ or the entanglement dimensionality of a system^21^. The alternative method to increase the entanglement dimensionality of a system is to use the LG modes. High-dimensional entanglement has recently attracted increasing attention in both fundamental and applied research in quantum mechanics. The use of these states can enhance quantum communication schemes by increasing their channel capacity and offering improved robustness against sophisticated eavesdropping attacks. However, one of the methods for generating higher order entangled states is using the OAM basis^22^. Recently, quantum logic gates also have been achieved experimentally^23^ and applied for generating high dimensional Bell States in this basis^24^. Although there are a lot of scientific reports on high dimensional photon states in OAM basis, it seems a deep fundamental research is necessary for atom-photon interaction in OAM basis, too. Moreover, we believe that the simulated atom-photon system can be a good source for high dimensional entangled states.

In this paper, we study the effect of intensity profile and OAM of light beams on the quantum entanglement in the three-level *V*-type quantum systems. In the first scheme, we consider *V*-type atomic system with two near-degenerate excited levels. In the presence of quantum coherence due to the spontaneous emission the transition of the electron between two excited states becomes possible and the *V*-type atomic system switches to the closed-loop configuration. In the second scheme, we consider two non-degenerate excited states and the quantum coherence between two upper levels is induced by applying a microwave field to the upper levels transition. Then, the optical properties of such systems depend on the OAM of the applied fields. We are interested in studying the entanglement of dressed atom and its spontaneous emission, using the LG fields with different OAMs. We use the von Neumann entropy as a measure of the degree of entanglement (DEM). It is shown that the DEM completely depends on the position of space points, so that the various points in the atomic vapor cell experience different DEM. Moreover, we find that the DEM is dependent on both magnitude and sign of the OAM of light beams. Then, the DEM can be controlled by the intensity profile, as well as the OAM of the applied fields. It is worth noting that the maximal value of DEM is obtained in the second scheme.

## Theoretical Framework

In general, an entangled quantum system consisting of two subsystems, *A* and *B*, is described by the reduced density matrix. The state of the entangled system is not a simple tensor product of the subsystems reduced density matrices, ${\rho }_{AB}\ne {\rho }_{A}\otimes {\rho }_{B}$. In thermodynamics, entropy is a fundamental physical quantity describing the degree of randomness of the system. Thus, we can represent the entanglement adopting the reduced quantum entropy. Various measures such as the reduced entropy of entanglement^25^, the relative entropy of entanglement^7^, entanglement of formation^26^ and entanglement of distillation^27^ have been introduced for DEM. Here, we use the definition of the von Neumann reduced entropy of entanglement. The von Neumann entropy, as a helpful quantity for calculating the DEM between the subsystems, is given by

1 $$S=-\,Tr(\rho ln\rho ),$$

where $\rho$ is the density matrix operator. It can be easily found that the von Neumann entropy vanishes for a bipartite system in pure state^28^. In 1970, Araki and Lieb demonstrated that the subsystems entropies satisfy in the triangle inequality^29^

2 $$|{S}_{A}(t)-{S}_{B}(t)|\le {S}_{AB}(t)\le |{S}_{A}(t)+{S}_{B}(t)|$$

at any time *t*. Here, ${S}_{AB}(t)$ is the total entropy of the composite system. Based on equation (2), for a atom-field system initially in a disentangled pure state, partial entropies of the field and the atom will be equal at all times after the interaction of two subsystems is switched on. Then, our information about any of the subsystems is an indication of the entanglement of the whole system. A decreasing partial entropy means that each subsystem evolves towards a pure quantum state, whereas in an initially pure system an increasing partial entropy drives the two components to lose their individuality and become entangled^30^. So the DEM for atom-field system would be

3 $$DEM(t)={S}_{A}={S}_{B}=-\,\sum _{i=1}^{N}\,{\lambda }_{i}ln{\lambda }_{i},$$

where *λ*_*i*_ is the reduced density matrix operator eigenvalues. The maximum value of DEM for a *N*-level quantum system is given by *lnN*, in which the population is uniformly distributed in the dressed states of the system^31,32^. Now, we are going to study the effect of applied LG fields on the atom-photon entanglement in two schemes of closed-loop three-level atomic systems.

## Three-Level *V*-Type Atomic Systems

**Figure 1:**
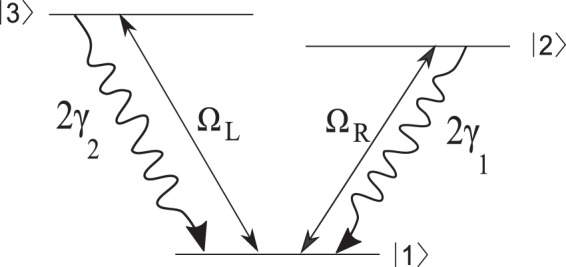
Schematics of the three-level $V$-type atomic system with two near-degenerate excited states $\mathrm{|2}\rangle$ and $\mathrm{|3}\rangle$ and a ground state $\mathrm{|1}\rangle$. $2{\gamma }_{1}$ and $2{\gamma }_{2}$ are the spontaneous emission rates from upper levels to lower level which are in the order of MHz. Two applied LG fields, ${{\rm{\Omega }}}_{L}$ and ${{\rm{\Omega }}}_{R}$, and the SGC make the proposed system a closed-loop system.

We assume an ensemble of three-level *V*-type atomic system with two near-degenerate excited states $\mathrm{|2}\rangle$ and $\mathrm{|3}\rangle$ and a ground state $\mathrm{|1}\rangle$, as shown in Fig. 1. As a realistic example, we consider the sodium ${D}_{2}$ line (${\mathrm{|3}}^{2}{S}_{\mathrm{1/2}},F=1\rangle \leftrightarrow {\mathrm{|3}}^{2}{P}_{\mathrm{3/2}}\rangle$); $\mathrm{|1}\rangle$, $\mathrm{|2}\rangle$, and $\mathrm{|3}\rangle$ correspond to sublevels ${\mathrm{|3}}^{2}{S}_{\mathrm{1/2}},F=1\rangle$, ${\mathrm{|3}}^{2}{P}_{\mathrm{3/2}},F^{\prime} =0\rangle$, and ${\mathrm{|3}}^{2}{P}_{\mathrm{3/2}},F^{\prime} =1\rangle$, respectively. Here, the upper levels are not exactly degenerated, however, if the energy difference of the upper levels is less than the natural line width, it can be considered as the near-degenerate levels and both transitions interact with a common vacuum state. Here the energy difference of upper levels is 15.8 MHz, which is less than the natural line width of the transition $(\,\sim \,61.35\,MHz)$. The spontaneous emission rates from two near-degenerate excited states $\mathrm{|2}\rangle$ and $\mathrm{|3}\rangle$ to the ground state is denoted by $2{\gamma }_{1}$ and $2{\gamma }_{2}$, respectively. In such a system, the spontaneous emission passing through the indistinguishable paths lead to the quantum interference called as spontaneously generated coherence (SGC). The transition $\mathrm{|1}\rangle \leftrightarrow \mathrm{|2}\rangle$ is driven by the right LG field with frequency ${\omega }_{R}$ and Rabi frequency ${{\rm{\Omega }}}_{R}={\overrightarrow{\mu }}_{12}\mathrm{.}{\overrightarrow{E}}_{R}/\hslash$, while the transition $\mathrm{|1}\rangle \leftrightarrow \mathrm{|3}\rangle$ is excited by the left LG field with frequency ${\omega }_{L}$ and Rabi frequency ${{\rm{\Omega }}}_{L}={\overrightarrow{\mu }}_{13}.{\overrightarrow{E}}_{L}/\hslash$. Here ${\overrightarrow{E}}_{R(L)}$ denotes the amplitude of right (left) LG field. The parameter ${\mu }_{ij}$ represents the induced dipole moment of the $|i\rangle \leftrightarrow |j\rangle$ transition.

The interaction Hamiltonian of the system under the electric-dipole and rotating-wave approximations can be written as

4 $${H}_{I}=-\,\hslash {{\rm{\Omega }}}_{R}{e}^{i{{\rm{\Delta }}}_{R}t}\mathrm{|2}\rangle \langle \mathrm{1|}-\hslash {{\rm{\Omega }}}_{L}{e}^{i{{\rm{\Delta }}}_{L}t}\mathrm{|3}\rangle \langle \mathrm{1|}+h\mathrm{.}c\mathrm{.,}$$

where ${{\rm{\Delta }}}_{R}={\omega }_{R}-{\omega }_{21}$ and ${{\rm{\Delta }}}_{L}={\omega }_{L}-{\omega }_{31}$ are the applied fields frequency detunings with respect to the atomic transition frequencies. The parameter ${\omega }_{ij}$ is the central frequency of $|i\rangle \leftrightarrow |j\rangle$ transition. Both applied fields are chosen as the LG fields and are given by

5 $$\begin{array}{c}{E}_{L}(r,\phi )={E}_{{0}_{L}}\frac{{w}_{G}}{\sqrt{|{l}_{L}|!}{w}_{LG}}{(\frac{\sqrt{2}r}{{w}_{LG}})}^{|{l}_{L}|}{e}^{-{r}^{2}/{w}_{LG}^{2}}{e}^{i{l}_{L}\phi },\\ {E}_{R}(r,\phi )={E}_{{0}_{R}}\frac{{w}_{G}}{\sqrt{|{l}_{R}|!}{w}_{LG}}{(\frac{\sqrt{2}r}{{w}_{LG}})}^{|{l}_{R}|}{e}^{-{r}^{2}/{w}_{LG}^{2}}{e}^{i{l}_{R}\phi },\end{array}$$

where ${E}_{{0}_{L}}$(${E}_{{0}_{R}}$) and ${l}_{L}$(${l}_{R}$) are the left(right) field amplitude and Orbital angular momentum, respectively. Gaussian and LG beam waists are denoted by ${w}_{G}$ and ${w}_{LG}$, respectively. Note that the beam waists of left and right applied fields are considered to be equal for enhancement the effect of their interaction with atomic system on the DEM. Using the von Neumann equation, the Bloch equations for such a system takes the form

6 $$\begin{array}{c}{\dot{\rho }}_{22}=-\,2{\gamma }_{1}{\rho }_{22}+i{{\rm{\Omega }}}_{R}^{\ast }{e}^{-i{\rm{\Delta }}t}{\rho }_{12}-i{{\rm{\Omega }}}_{R}{e}^{i{\rm{\Delta }}t}{\rho }_{21}-\eta \sqrt{{\gamma }_{2}{\gamma }_{1}}({\rho }_{32}+{\rho }_{23}),\\ {\dot{\rho }}_{33}=-\,2{\gamma }_{2}{\rho }_{33}+i{{\rm{\Omega }}}_{L}^{\ast }{\rho }_{13}-i{{\rm{\Omega }}}_{L}{\rho }_{31}-\eta \sqrt{{\gamma }_{2}{\gamma }_{1}}({\rho }_{32}+{\rho }_{23}),\\ {\dot{\rho }}_{12}=(\,-\,{\gamma }_{1}+i({{\rm{\Delta }}}_{R}+{\rm{\Delta }})){\rho }_{12}+i{{\rm{\Omega }}}_{L}{\rho }_{32}+i{{\rm{\Omega }}}_{R}{e}^{i{\rm{\Delta }}t}({\rho }_{22}-{\rho }_{11})-\eta \sqrt{{\gamma }_{2}{\gamma }_{1}}{\rho }_{13},\\ {\dot{\rho }}_{13}=(\,-\,{\gamma }_{2}+i{{\rm{\Delta }}}_{L}){\rho }_{13}+i{{\rm{\Omega }}}_{R}{e}^{i{\rm{\Delta }}t}{\rho }_{23}+i{{\rm{\Omega }}}_{L}({\rho }_{33}-{\rho }_{11})-\eta \sqrt{{\gamma }_{2}{\gamma }_{1}}{\rho }_{12},\\ {\dot{\rho }}_{23}=(\,-\,({\gamma }_{1}+{\gamma }_{2})+i({{\rm{\Delta }}}_{L}-{{\rm{\Delta }}}_{R}-{\rm{\Delta }})){\rho }_{23}-i{{\rm{\Omega }}}_{L}{\rho }_{21}+i{{\rm{\Omega }}}_{R}^{\ast }{e}^{-i{\rm{\Delta }}t}{\rho }_{13}-\eta \sqrt{{\gamma }_{2}{\gamma }_{1}}({\rho }_{22}+{\rho }_{33}),\\ {\dot{\rho }}_{11}=-\,({\dot{\rho }}_{22}+{\dot{\rho }}_{33}),\end{array}$$

where ${\rm{\Delta }}={{\rm{\Delta }}}_{R}-{{\rm{\Delta }}}_{L}={\omega }_{R}-{\omega }_{L}$. The parameter $\eta$ in equation (6) stands for the SGC strength.

We now introduce the dressed states generated by applied fields which are useful for understanding the optical properties of the system. The physics of the entanglement can be explained via the population distribution of the dressed states. For the proposed model, the dressed states in the absence of spontaneous emissions are given by

7 $$\begin{array}{c}|N\rangle =\frac{1}{\sqrt{|{{\rm{\Omega }}}_{L}{|}^{2}+|{{\rm{\Omega }}}_{R}{|}^{2}}}({{\rm{\Omega }}}_{L}|2\rangle -{{\rm{\Omega }}}_{R}|3\rangle ),\\ |C\rangle =\frac{1}{\sqrt{|{{\rm{\Omega }}}_{L}{|}^{2}+|{{\rm{\Omega }}}_{R}{|}^{2}}}({{\rm{\Omega }}}_{R}^{\ast }|2\rangle +{{\rm{\Omega }}}_{L}^{\ast }|3\rangle ),\\ |0\rangle =|1\rangle .\end{array}$$

The dressed states population can be obtained as

8 $$\begin{array}{c}{\rho }_{NN}=|N\rangle \langle N|=\frac{1}{|{{\rm{\Omega }}}_{L}{|}^{2}+|{{\rm{\Omega }}}_{R}{|}^{2}}(|{{\rm{\Omega }}}_{L}{|}^{2}{\rho }_{22}+|{{\rm{\Omega }}}_{R}{|}^{2}{\rho }_{33}-{{\rm{\Omega }}}_{L}{{\rm{\Omega }}}_{R}^{\ast }{\rho }_{23}-{{\rm{\Omega }}}_{L}^{\ast }{{\rm{\Omega }}}_{R}{\rho }_{32}),\\ {\rho }_{CC}=|C\rangle \langle C|=\frac{1}{|{{\rm{\Omega }}}_{L}{|}^{2}+|{{\rm{\Omega }}}_{R}{|}^{2}}(|{{\rm{\Omega }}}_{R}{|}^{2}{\rho }_{22}+|{{\rm{\Omega }}}_{L}{|}^{2}{\rho }_{33}+{{\rm{\Omega }}}_{L}{{\rm{\Omega }}}_{R}^{\ast }{\rho }_{23}+{{\rm{\Omega }}}_{L}^{\ast }{{\rm{\Omega }}}_{R}{\rho }_{32}),\\ {\rho }_{00}=|0\rangle \langle 0|={\rho }_{11}.\end{array}$$

**Figure 2:**
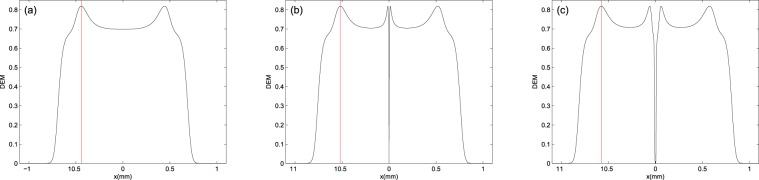
Steady-state behavior of the DEM versus $x$ for (**a**) ${l}_{L}={l}_{R}=0$, (**b**) ${l}_{L}={l}_{R}=1$ and (**c**) ${l}_{L}={l}_{R}=2$. Used parameters are ${\gamma }_{1}={\gamma }_{2}=\gamma$, $\eta =0.99$, ${{\rm{\Omega }}}_{{0}_{L}}=7\gamma$, ${\Omega }_{{0}_{R}}=9\gamma$, ${w}_{G}=1\,mm$, ${w}_{LG}=270\,\mu m$ and ${{\rm{\Delta }}}_{L}={{\rm{\Delta }}}_{R}=0$. Red lines indicate the maximum of DEM position in all panels.

Now, we are interested in studying the DEM for *V*-type atomic system by numerically solving equations (3) and (6). It is assumed that the multi-photon resonance condition is fulfilled. All frequency parameters are scaled by ${\gamma }_{1}$, which is in the order of MHz for the chosen atomic system. We consider different intensity profiles, i.e., Gaussian and LG modes for the applied fields and investigate steady-state behavior of the DEM in different points of atomic vapor cell. Figure 2 shows the DEM as a function of *x* for the applied Gaussian fields, (a) ${l}_{L}={l}_{R}=0$, first mode (b) ${l}_{L}={l}_{R}=1$ and second mode of applied LG fields (c) ${l}_{L}={l}_{R}=2$. Used parameters are ${\gamma }_{1}={\gamma }_{2}=\gamma$, $\eta =0.99$, ${{\rm{\Omega }}}_{{0}_{L}}=7\gamma$, ${{\rm{\Omega }}}_{{0}_{R}}=9\gamma$, ${w}_{G}=1\,mm$, ${w}_{LG}=270\,\mu m$ and ${{\rm{\Delta }}}_{L}={{\rm{\Delta }}}_{R}=0$, multi-photon resonance condition. Red lines indicate the maximum of DEM positions in panels (a)–(c). In panel (a), it can be easily seen that the DEM changes from zero to a maximum value, for different values of *x*. The DEM vanishes where the beams’ intensity becomes zero. Panels (b) and (c) shows that the zero intensity in the center of LG profile implies a disentanglement in the center of LG modes. So, the spatially dependent behavior of DEM is related to the intensity profile of two applied fields. Note that the effect of OAMs’ sign on the DEM is not seen in one dimension calculation.

**Figure 3:**
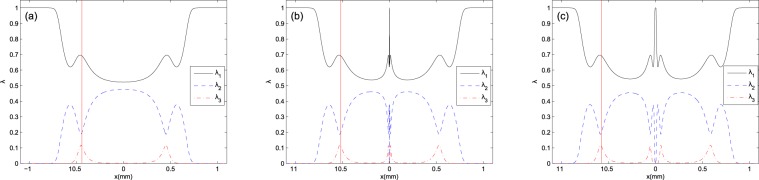
Steady-state behavior of density matrix eigenstates versus $x$ for (**a**) ${l}_{L}={l}_{R}=0$, (**b**) ${l}_{L}={l}_{R}=1$ and (**c**) ${l}_{L}={l}_{R}=2$. Solid, dashed and dash-dotted curves show the first, second and third eigenstates, respectively. The same parameters as in Fig. 2 are used in all panels. Red line, in each of panels, corresponds to the position of maximum of DEM.

A physical meaning of entanglement lies on the dressed states conception. For explanation of DEM behavior in the presence of spontaneous emissions, we calculate the density matrix operator eigenvalues which describe the dressed states population in the presence of spontaneous emmision. The uniform population distribution of the dressed states induce the maximal entanglement to the atomic system. By numerically solving equation (6) and diagonalizing the density matrix, the dressed states population can be obtained as a function of *x*. We would like to investigate the dressed states population in different points of the atomic vapor cell. In the following, Fig. 3 shows the dressed states population versus *x* corresponding to Fig. 2. As seen in all panels of Fig. 3, the population is distributed only in two states around $x=0$ and the DEM value reaches to $ln2$, while a part of population transfers to the third state at the maximum point of DEM and the population is nearly uniformly distributed in the three dressed states, with respect to the population distribution at $x=0$.

**Figure 4:**
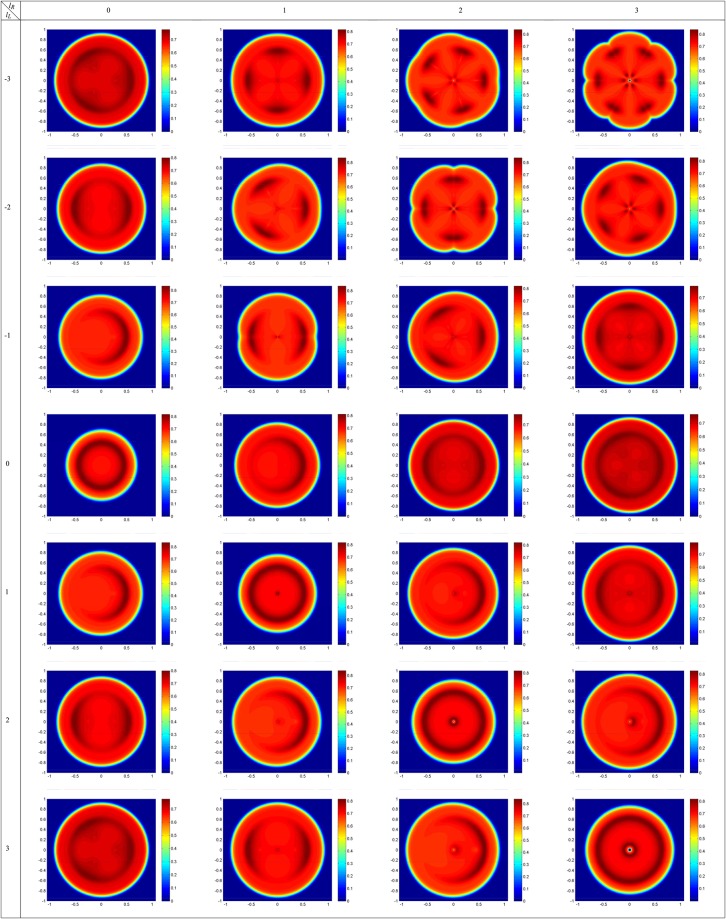
DEM density plots as a function of $x$ and $y$ for different modes of applied fields with ${l}_{L}=-\,\mathrm{3,}\,\mathrm{...,}\,3$ and ${l}_{R}=\mathrm{0,}\,\mathrm{...,}\,3$. Size of each density plot is $2\,mm\,\times \,2\,mm$ in which horizontal and vertical axes are $x$ and $y$ axes, respectively. Used parameters are ${\gamma }_{1}={\gamma }_{2}=\gamma$, $\eta =0.99$, ${{\rm{\Omega }}}_{{0}_{L}}=7\gamma$, ${{\rm{\Omega }}}_{{0}_{R}}=9\gamma$, ${w}_{G}=1\,mm$, ${w}_{LG}=270\,\mu m$ and ${{\rm{\Delta }}}_{L}={{\rm{\Delta }}}_{R}=0$.

Note that in one dimension calculation, the effect of the OAM of light beams on the atom-photon entanglement does not take into account, so, we have to continue our calculations in two dimensions to include the contribution of the OAM of applied LG fields. According to equation (5), the OAM of light appears as a phase term, ${e}^{il\phi }$. It is expected that the optical properties of the *V*-type atomic system depend on the OAM of light beam in closed-loop configuration. In Fig. 4, we depict the DEM density plots as a function of *x* and $y$ for different modes of applied fields with ${l}_{L}=-\,3,\ldots ,3$ and ${l}_{R}=0,\ldots ,3$. Used parameters are ${\gamma }_{1}={\gamma }_{2}=\gamma$, $\eta =0.99$, ${{\rm{\Omega }}}_{{0}_{L}}=7\gamma$, ${{\rm{\Omega }}}_{{0}_{R}}=9\gamma$, ${w}_{G}=1\,mm$, ${w}_{LG}=270\,\mu m$ and ${{\rm{\Delta }}}_{L}={{\rm{\Delta }}}_{R}=0$. There are $28$ panels for different choices of LG modes of applied fields. For ${l}_{L}={l}_{R}=0$, the DEM changes from zero to its maximum value in a determined radius and decreases to nearly maximal value of DEM of two-level system, $ln2$. However, for equal OAM modes of two applied LG fields, ${l}_{L}={l}_{R}=l$, the DEM value has a central symmetry with a disentanglement point at the origin coordinates due to the zero intensity of applied fields. These results in the $x$ direction are in good agreement with one dimensional results of Fig. 2. Here, we show that the DEM behavior completely depends on the OAM of applied fields in two dimensions. The local maximum DEM ring switches to $2l$ maximum DEM regions when the rotation direction of wavefront is turned to counter-clockwise, ${l}_{L}=-\,{l}_{R}=l$. The obtained results are in good agreement with the presented petal-like patterns in references^33^ and^34^ in which, it was shown that the superposition pattern of two LG beams with $l$ of opposite sign has a symmetric structure with $2l$ petals. The DEM profile is similar to the interference pattern of two applied LG fields. Moreover, we extend our calculations for different LG modes and it can be generally seen that the number of local maximum peaks equals $|{l}_{L}-{l}_{R}|$ in all panels. So, the atom-photon entanglement pattern can be controlled by OAM of light beams.

**Figure 5:**
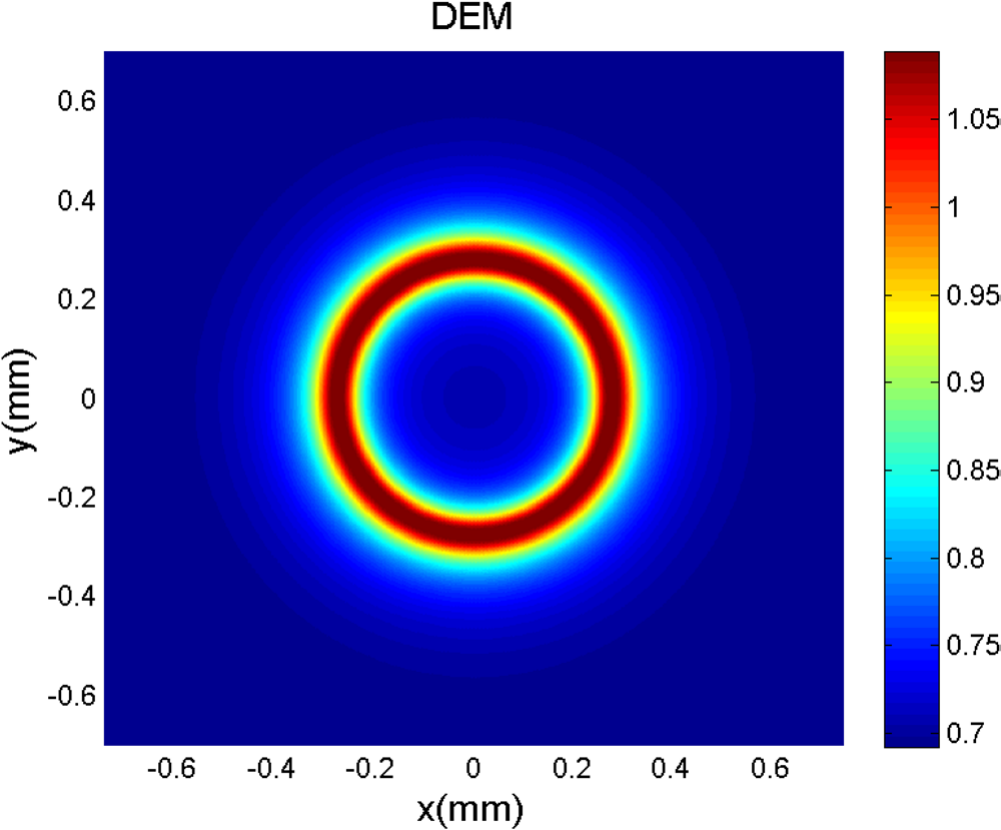
Density plot of the DEM versus $x$ and $y$, beyond multi-photon resonance condition, when two applied fields are considered as a plane-wave (right field) and a Gaussian (left field). Used parameters for the applied fields are ${{\rm{\Omega }}}_{R}=9\gamma$, ${{\rm{\Omega }}}_{{0}_{L}}=7\gamma$, ${w}_{G}=1\,mm$ and ${\rm{\Delta }}=\gamma$. The atomic parameters are ${\gamma }_{1}={\gamma }_{2}=\gamma$ and $\eta =0.99$.

**Figure 6:**
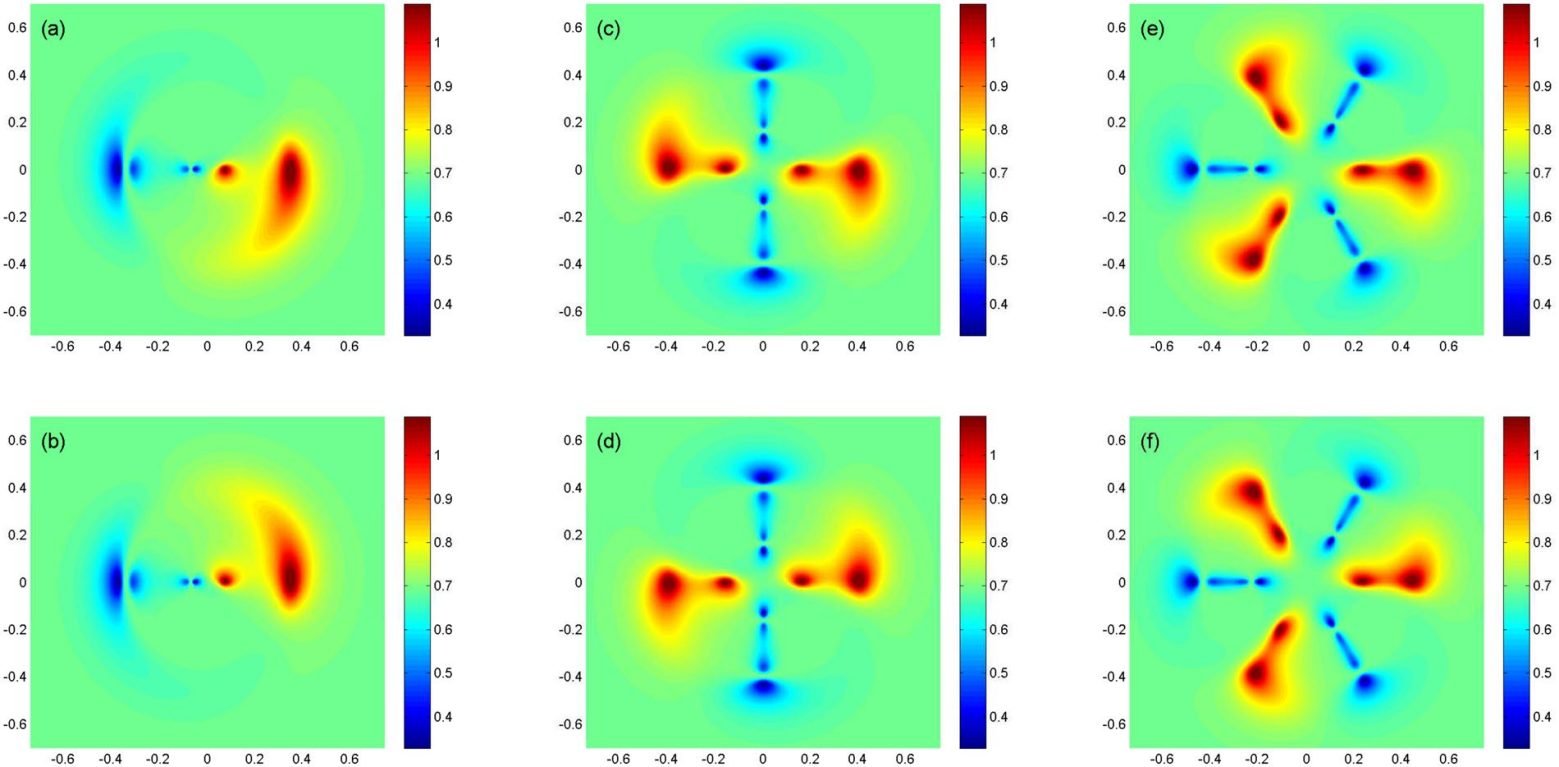
DEM density plots as a function of $x$ and $y$ for a plane-wave laser as the right field and a LG field as the left field with different modes, ${l}_{L}=1$ and $-1$, (**a**,**b**), ${l}_{L}=2$ and $-2$, (**c**,**d**), ${l}_{L}=3$ and $-3$, (**e**,**f**), beyond multi-photon resonance condition. Size of each density plot is $1.4\,mm\,\times \,1.4\,mm$ in which horizontal and vertical axes are $x$ and $y$ axes, respectively. The applied fields parameters are ${{\rm{\Omega }}}_{R}=9\gamma$, ${{\rm{\Omega }}}_{{0}_{L}}=7\gamma$, ${w}_{LG}=270\,\mu m$ and ${\rm{\Delta }}=\gamma$. The atomic properties are chosen to be same as in Fig. 5.

Now, we are going to investigate the DEM behavior of the system beyond multi-photon resonance condition, ${\rm{\Delta }}\ne 0$. However, we drop the explicit time dependent phase factors in equation (6). Then, our results are valid only near multi-photon resonance condition. The right (left) field is considered as a plane-wave (LG field). In Fig. 5, we display the DEM density plot as a function of $x$ and $y$ in different points of the cross-section of atomic vapor cell for a Gaussian left field, ${l}_{L}\mathrm{=0}$. Used parameters for the applied fields are assumed to be ${{\rm{\Omega }}}_{R}=9\gamma$, ${{\rm{\Omega }}}_{{0}_{L}}=7\gamma$, ${w}_{G}=1\,mm$ and ${\rm{\Delta }}=\gamma$, beyond multi-photon resonance condition. The atomic parameters are ${\gamma }_{1}={\gamma }_{2}=\gamma$ and $\eta =0.99$. It is shown that the DEM tends to its maximal value, $ln3$, in a special radius which depends on the characteristics of the Gaussian field. The DEM density plots for the different modes of LG field are plotted versus $x$ and $y$, as shown in Fig. 6. The applied fields parameters are ${{\rm{\Omega }}}_{R}=9\gamma$, ${{\rm{\Omega }}}_{{0}_{L}}=7\gamma$, ${w}_{LG}=270\,\mu m$ and ${\rm{\Delta }}=\gamma$. The atomic properties are chosen to be same as in Fig. 5. Figure 6(a) shows the DEM variations for the first mode of LG field, ${l}_{L}=1$. The DEM behavior, specially around maximal DEM regions, is very similar to spiral-shaped fringes due to the interference of first mode of LG light beam with a plane-wave^18,34^. In Fig. 6(b) it is clearly seen that the DEM behavior for the negative OAM of the first mode of LG field is in good agreement with the rotation direction of spiral arms of LG field and plane-wave interferogram. We repeat our numerical calculations for positive and negative OAMs of the second and third modes of LG left field, ${l}_{L}=\mathrm{2,}-\,\mathrm{2,}\,\mathrm{3,}-\,3$ in panels (c)–(f), respectively. An investigation on Fig. 6(c–f) shows that the DEM patterns are similar to the interference pattern of different modes of LG beam with the plane-wave. Therefore, the spatially dependent DEM can be controlled either magnitude or sign of the OAM of the applied LG field.

## Closed-Loop Three-Level *V*-Type Atomic Systems

**Figure 7:**
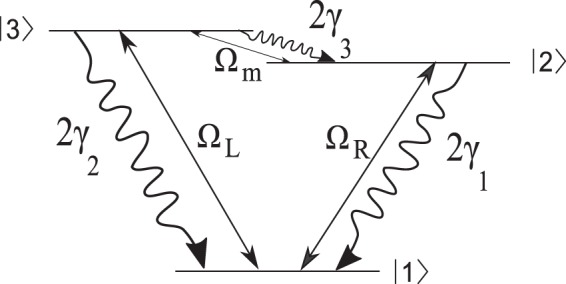
Schematics of the closed-loop three-level $V$-type atomic system with two non-degenerate excited states $\mathrm{|2}\rangle$ and $\mathrm{|3}\rangle$ and a ground state $\mathrm{|1}\rangle$ which can be prepared in the Rb Rydberg atoms. The spontaneous emission rate related to $\mathrm{|2}\rangle \leftrightarrow \mathrm{|3}\rangle$ transition is denoted by $2{\gamma }_{3}$; $2{\gamma }_{1}$ and $2{\gamma }_{2}$ are the spontaneous emission rates from upper levels to lower level which are in the order of MHz. Two applied LG fields, ${{\rm{\Omega }}}_{L}$ and ${{\rm{\Omega }}}_{R}$ and one planar microwave field, ${{\rm{\Omega }}}_{m}$, establish a closed-loop atomic system.

In this section, we propose an ensemble of three-level *V*-type atomic system with two non-degenerate excited states and apply a planar microwave field, ${{\rm{\Omega }}}_{m}$, to the $\mathrm{|2}\rangle \leftrightarrow \mathrm{|3}\rangle$ transition and two other fields shown in Fig. 7, which can be prepared in the Rb Rydberg atoms^35^.

Using the von Neumann equation and the interaction Hamiltonian of the system, the Bloch equations for the system are obtained by

9 $$\begin{array}{c}{\dot{\rho }}_{22}=-\,2{\gamma }_{1}{\rho }_{22}+2{\gamma }_{3}{\rho }_{33}+i{{\rm{\Omega }}}_{R}^{\ast }{\rho }_{12}-i{{\rm{\Omega }}}_{R}{\rho }_{21}+i{{\rm{\Omega }}}_{m}{\rho }_{32}-i{{\rm{\Omega }}}_{m}^{\ast }{\rho }_{23},\\ {\dot{\rho }}_{33}=-\,2({\gamma }_{2}+{\gamma }_{3}){\rho }_{33}+i{{\rm{\Omega }}}_{L}^{\ast }{e}^{-i{\rm{\Delta }}t}{\rho }_{13}-i{{\rm{\Omega }}}_{L}{e}^{i{\rm{\Delta }}t}{\rho }_{31}-i{{\rm{\Omega }}}_{m}{\rho }_{32}+i{{\rm{\Omega }}}_{m}^{\ast }{\rho }_{23},\\ {\dot{\rho }}_{12}=(\,-\,{\gamma }_{1}+i{{\rm{\Delta }}}_{R}){\rho }_{12}+i{{\rm{\Omega }}}_{L}{e}^{i{\rm{\Delta }}t}{\rho }_{32}+i{{\rm{\Omega }}}_{R}({\rho }_{22}-{\rho }_{11})-i{{\rm{\Omega }}}_{m}^{\ast }{\rho }_{13},\\ {\dot{\rho }}_{13}=(\,-\,({\gamma }_{2}+{\gamma }_{3})+i({{\rm{\Delta }}}_{L}-{\rm{\Delta }})){\rho }_{13}+i{{\rm{\Omega }}}_{R}{\rho }_{23}+i{{\rm{\Omega }}}_{L}{e}^{i{\rm{\Delta }}t}({\rho }_{33}-{\rho }_{11})-i{{\rm{\Omega }}}_{m}{\rho }_{12},\\ {\dot{\rho }}_{23}=(\,-\,({\gamma }_{1}+{\gamma }_{2}+{\gamma }_{3})+i{{\rm{\Delta }}}_{m}){\rho }_{23}-i{{\rm{\Omega }}}_{L}{e}^{i{\rm{\Delta }}t}{\rho }_{21}+i{{\rm{\Omega }}}_{R}^{\ast }{\rho }_{13}+i{{\rm{\Omega }}}_{m}({\rho }_{33}-{\rho }_{22}),\\ {\dot{\rho }}_{11}=-\,({\dot{\rho }}_{22}+{\dot{\rho }}_{33}),\end{array}$$

where $${\rm{\Delta }}={{\rm{\Delta }}}_{L}-{{\rm{\Delta }}}_{R}-{{\rm{\Delta }}}_{m}$$.

**Figure 8:**
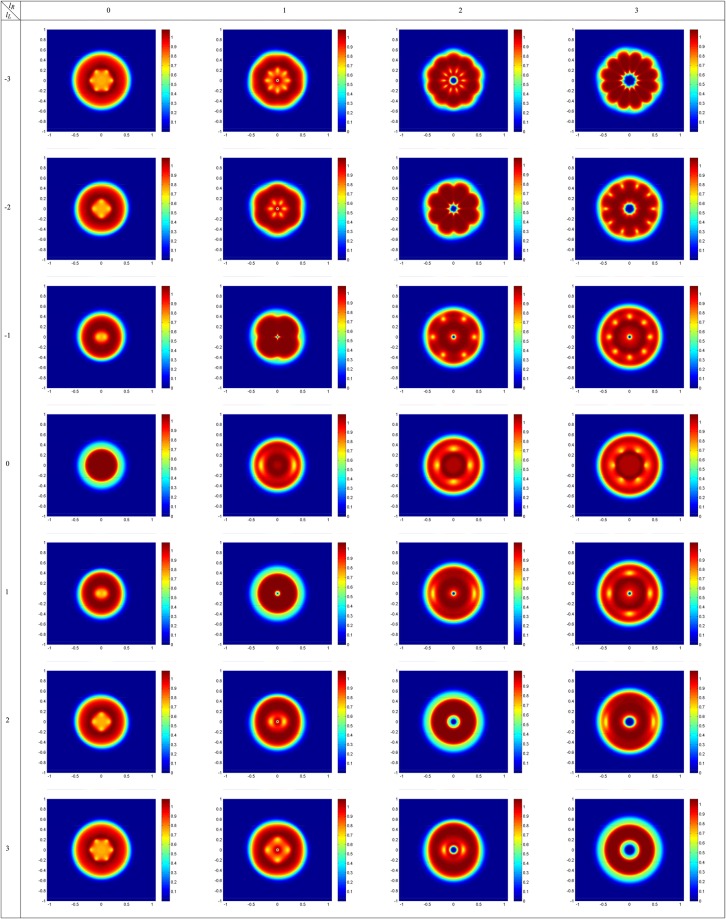
DEM density plots as a function of $x$ and $y$ for different modes of applied fields with ${l}_{L}=-\,\mathrm{3,}\,\mathrm{...,}\,3$ and ${l}_{R}=\mathrm{0,}\,\mathrm{...,}\,3$. Size of each density plot is $2\,mm\,\times \,2\,mm$ in which horizontal and vertical axes are $x$ and $y$ axes, respectively. Three applied fields characteristics are chosen to be ${{\rm{\Omega }}}_{{0}_{L}}=7\gamma$, ${{\rm{\Omega }}}_{{0}_{R}}=9\gamma$, ${{\rm{\Omega }}}_{m}=7\gamma$, ${w}_{G}=1\,mm$, ${w}_{LG}=270\,\mu m$ and ${{\rm{\Delta }}}_{L}={{\rm{\Delta }}}_{R}={{\rm{\Delta }}}_{m}=0$. The atomic system parameters are ${\gamma }_{1}={\gamma }_{2}=\gamma$ and ${\gamma }_{3}=2\gamma$.

Here, we are going to show the behavior of the DEM in a closed-loop three-level $V$-type atomic system using equations (3) and (9). A planar microwave field is applied to the $\mathrm{|2}\rangle \leftrightarrow \mathrm{|3}\rangle$ transition. It is assumed that the multi-photon resonance condition is fulfilled. Figure 8 presents the density plots of DEM versus $x$ and $y$ for different modes of applied fields with ${l}_{L}=-\,\mathrm{3,}\,\mathrm{...,}\,3$ and ${l}_{R}=\mathrm{0,}\,\mathrm{...,}\,3$. Size of each density plot is $2\,mm\,\times \,2\,mm$ in which horizontal and vertical axes are $x$ and $y$ axes, respectively. The microwave Rabi frequency, relaxation rate between two excited states and detuning of the microwave field frequency are considered to be ${{\rm{\Omega }}}_{m}=7\gamma$, ${\gamma }_{3}=2\gamma$ and ${{\rm{\Delta }}}_{m}=0$, respectively. Other used parameters are ${\gamma }_{1}={\gamma }_{2}=\gamma$, ${{\rm{\Omega }}}_{{0}_{L}}=7\gamma$, ${{\rm{\Omega }}}_{{0}_{R}}=9\gamma$, ${w}_{G}=1\,mm$, ${w}_{LG}=270\,\mu m$ and ${{\rm{\Delta }}}_{L}={{\rm{\Delta }}}_{R}=0$. It is shown that the maximal entanglement can be obtained in different regions of atomic vapor cell by applying a planar microwave field instead of the SGC effect. Two applied Gaussian fields, ${l}_{L}={l}_{R}=0$, create maximal value of DEM in a central circular region of atomic vapor cell. Now, let us refer to the DEM density plots in which two applied LG fields have equal OAMs, ${l}_{L}={l}_{R}=l$. A comparison between the related panels shows that the region of disentanglement has been grown for larger OAMs. It is worth to note that for LG modes with opposite sign OAMs, ${l}_{L}=-\,{l}_{R}=l$, the maximal DEM doughnut-like region splits to $4l$ symmetric segments. Other $21$ planes in Fig. 8 show the DEM behavior in different points of the atomic vapor cell for different choices of OAM of two applied fields. An investigation on all panels of Fig. 8 confirms that the DEM patterns follow a common model which are extremely dependent on OAM and have $\mathrm{2|}{l}_{L}-{l}_{R}|$ maximal regions.

## Conclusion

In conclusion, we studied quantum entanglement between an ensemble of three-level atomic system and its spontaneous emissions. In the first scheme, we investigated the steady-state behavior of the DEM in a three-level $V$-type atomic system with the SGC effect under the multi-photon resonance condition. We found an intriguing result which illustrates an explicit dependency between the DEM density plot and the OAM of LG light beams. Moreover, we showed that the DEM density plot pattern is corresponding to the interference pattern of the two applied fields. In the second scheme, a microwave plane field was applied to the non-degenerate upper levels transition which leads to the maximal DEM. It was demonstrated that the number of maximal entanglement peaks is determined by the OAM of the applied LG fields. Both disentanglement and maximal DEM were simultaneously obtained in the second scheme. The presented results can be used in optical communications and information storage via preparing high-dimensional Hilbert space.

## References

[1] Brassard G, Chuang I, Lloyd S, Monroe C (1998). Quantum computing. Proc. Natl. Acad. Sci. USA.

[2] Horodecki R, Horodecki P, Horodecki M, Horodecki K (2009). Quantum entanglement. Rev. Mod. Phys..

[3] Ficek, Z. & Swain, S. *Quantum Coherence and Interference; Theory and Experiments* (Springer, 2004).

[4] Freedman SJ, Clauser JF (1972). Experimental test of local hidden variables theories. Phys. Rev. Lett..

[5] Bennett CH (1993). Teleporting an unknown quantum state via dual classical and Einstein-Podolsky-Rosen channels. Phys. Rev. Lett..

[6] Ekert AK (1991). Quantum cryptography based on Bell’s theorem. Phys. Rev. Lett..

[7] Bennett CH, DiVincenzo DP, Smolin JA, Wootters WK (1996). Mixed-state entanglement and quantum error correction. Phys. Rev. A.

[8] Benenti, G., Casati, G. & Strini, G. *Principles of Quantum Computation and Information; vol 1: Basic Concepts* (World Scientific, 2004).

[9] Beth RA (1936). Mechanical detection and measurement of the angular momentum of light. Phys. Rev..

[10] Allen L, Beijersbergen MW, Spreeuw RJC, Woerdman JP (1992). Orbital angular momentum of light and the transformation of Laguerre-Gaussian laser modes. Phys. Rev. A.

[11] Anupriya J, Ram N, Pattabiraman M (2010). Hanle electromagnetically induced transparency and absorption resonances with a Laguerre Gaussian beam. Phys. Rev. A.

[12] Chanu SR, Singh AK, Brun B, Pandey K, Natarajan V (2011). Subnatural linewidth in a strongly-driven degenerate two-level system. Opt. Commun..

[13] Akin TG, Krzyzewski SP, Marino AM, Abraham ERI (2015). Electromagnetically induced transparency with Laguerre-Gaussian modes in ultracold rubidium. Opt. Commun..

[14] Radwell N, Clark TW, Piccirillo B, Barnett SM, Franke-Arnold S (2015). Spatially dependent electromagnetically induced transparency. Phys. Rev. Lett..

[15] Kazemi SH, Mahmoudi M (2016). Multi-photon resonance phenomena using Laguerre-Gaussian beams. J. Phys. B: At. Mol. Opt. Phys..

[16] Kazemi SH, Ghanbari S, Mahmoudi M (2017). Trap split with Laguerre-Gaussian beams. J. Opt..

[17] Amini Sabegh Z, Maleki MA, Mahmoudi M (2017). Phase-controlled electromagnetically induced focusing in a closed-loop atomic system. J. Opt. Soc. Am. B.

[18] Padgett M, Courtial J, Allen L (2004). Light’s orbital angular momentum. Phys. Today.

[19] Mahmoudi M, Evers J (2006). Light propagation through closed-loop atomic media beyond the multiphoton resonance condition. Phys. Rev. A.

[20] Greenberger DM, Horne MA, Shimony A, Zeilinger A (1990). Bell’s theorem without inequalities. Am. J. Phys..

[21] Krenn M (2014). Generation and confirmation of a (100 × 100)-dimensional entangled quantum system. Proc. Natl. Acad. Sci. USA.

[22] Mair A, Vaziri A, Weihs G, Zeilinger A (2001). Entanglement of the orbital angular momentum states of photons. Nature.

[23] Babazadeh A (2017). High-dimensional single-photon quantum gates: Concepts and experiments. Phys. Rev. Lett..

[24] Wang F (2017). Generation of the complete four-dimensional Bell basis. Optica.

[25] Vedral V, Plenio MB, Rippin MA, Knight PL (1997). Quantifying entanglement. Phys. Rev. Lett..

[26] Audenaert K, Verstraete F, Moor BDe (2001). Variational characterizations of separability and entanglement of formation. Phys. Rev. A.

[27] Lo HK, Popescu S (2001). Concentrating entanglement by local actions: Beyond mean values. Phys. Rev. A.

[28] Phoenix SJD, Knight PL (1988). Fluctuations and entropy in models of quantum optical resonance. Ann. Phys..

[29] Araki H, Lieb EH (1970). Entropy inequalities. Commun. Math. Phys..

[30] Phoenix SJD, Knight PL (1991). Establishment of an entangled atom-field state in the Jaynes-Cummings model. Phys. Rev. A.

[31] Phoenix SJD, Knight PL (1991). Comment on Collapse and revival of the state vector in the Jaynes-Cummings model: An example of state preparation by a quantum apparatus. Phys. Rev. Lett..

[32] Abazari M, Mortezapour A, Mahmoudi M, Sahrai M (2011). Phase-controlled atom-photon entanglement in a three-level *V*-type atomic system via spontaneously generated coherence. Entropy.

[33] Li Y, Zhou ZY, Ding DS, Shi BS (2015). Sum frequency generation with two orbital angular momentum carrying laser beams. J. Opt. Soc. Am. B.

[34] Vickers J, Burch M, Vyas R, Singh S (2008). Phase and interference properties of optical vortex beams. J. Opt. Soc. Am. A.

[35] Tate DA, Gallagher TF (2018). Microwave-optical two-photon excitation of Rydberg states. Phys. Rev. A.

